# Evolutionary perspective of the CAG/CAA interplay coding for pure polyglutamine stretches in proteins

**DOI:** 10.1093/nargab/lqaf075

**Published:** 2025-06-09

**Authors:** Antonio Moreno-Rodríguez, Antonio J Pérez-Pulido, Pablo Mier

**Affiliations:** Andalusian Centre for Developmental Biology (CABD, UPO-CSIC-JA), Faculty of Experimental Sciences (Genetics Area), Universidad Pablo de Olavide, 41013 Seville, Spain; Andalusian Centre for Developmental Biology (CABD, UPO-CSIC-JA), Faculty of Experimental Sciences (Genetics Area), Universidad Pablo de Olavide, 41013 Seville, Spain; Andalusian Centre for Developmental Biology (CABD, UPO-CSIC-JA), Faculty of Experimental Sciences (Genetics Area), Universidad Pablo de Olavide, 41013 Seville, Spain

## Abstract

Polyglutamine regions appear in many eukaryotic proteins. Most research on these stretches has focused on humans and primates. We wanted to check whether patterns in their codon usage are shared across a wide taxonomic range. Protein-coding transcripts from 30 eukaryotic model species were searched for stretches of consecutive glutamine codons (CAA/CAG). Most species have higher CAG proportion in longer stretches, except fishes, which either reduced or kept a stable CAG use. CAA codons are located closer to the C-terminal side of the stretches in plants, invertebrates, and tetrapods; fungi showed no bias and fishes showed the opposite. Many tetrapods have codons flanking pure CAG stretches that hint at a mutational control of repeat growth. However, the maximum number of consecutive identical codons within the polyglutamine stretches in most species followed random expectations, with fishes as a main exception. We detected shared patterns in codon usage and position across taxonomically distant species, yet each group retained unique traits. Internal CAA position and external flanking codons both seemed to slow pure CAG expansion. Overall, a mix of random processes and species-specific factors drives how glutamine repeats are shaped and maintained in evolution.

## Introduction

Polyglutamine (polyQ) stretches, regions of consecutive glutamine residues in protein sequences, occur frequently across eukaryotic proteomes [1]. They belong to a broader category of low-complexity regions, characterized by repetitive, compositionally simple amino acid sequences and rapid evolution [2]. Glutamine residues are encoded by two synonymous codons: CAA and CAG. Although synonymous, these codons differ in genomic stability, with CAG repeats being more prone to expansion through a replication slippage mechanism [3]. CAA interruptions have been described to increase the fidelity of polymerase chain reaction amplification of CAG repeated sequences [4]. In some cases, replication slippage may extend the polyQ stretches resulting in pathogenically long stretches [5]. In humans, expansions of glutamine repeats are associated with severe neurodegenerative diseases, including Huntington’s disease, spinocerebellar ataxias, spinal and bulbar muscular atrophy, and dentatorubral-pallidoluysian atrophy [6–11]. Interestingly, CAA interruptions of the CAG repeat in the ATXN2 protein, whose expansion is associated with spinocerebellar ataxia 2, are linked to amyotrophic lateral sclerosis [12]. Despite substantial interest in pathological expansions, less attention has been given to polyQ evolution in nonpathogenic contexts.

Beyond codon composition, in humans it has been described that surrounding amino acids affect the structural stability of polyQ regions [13]. Proline-rich sequences immediately adjacent to polyQ stretches, for example, have been linked to reduced aggregation potential, leading to the formation of polyproline type II (PPII) helices that prevent toxic β-sheet structures typical in expanded polyQ-associated diseases [14, 15]. In other taxa, equivalent structural constraints likely exist but may involve different amino acids, reflecting selective pressures imposed by organism-specific functional requirements or mutational processes.

Most research into polyQ regions has been restricted to mammals, particularly humans and primates [16–18]. Comparative analyses that take into account broader evolutionary distances remain rare [19–21]. Therefore, it is unclear whether observed human-specific patterns, such as biases in codon usage or structural constraints, apply across eukaryotes.

In this study, we examined codon usage, position, and amino acid context of polyQ stretches in protein-coding transcripts from 30 diverse eukaryotic model species. These include plants, fungi, invertebrates, fishes, and tetrapods, chosen to reflect broad taxonomic representation and guarantee balanced comparisons. We did not use any prokaryotic species due to the low prevalence of glutamine stretches in their proteomes. By comparing such diverse organisms, we aimed to uncover general evolutionary patterns in polyQ regions and understand how different taxonomic groups maintain and stabilize these sequences.

## Materials and methods

### Data retrieval

We downloaded the complete set of protein-coding transcripts from 30 eukaryotic model species from the FTP sites of the Ensembl database release 113 [22], and the release 60 of EnsemblPlants, EnsemblFungi, and EnsemblMetazoa [23]. Species were selected based on their use as model organisms, as well as to maintain a balance between different taxonomic groups in Eukarya. For space purposes, each species name is shortened in the manuscript to a three-letter code. Information was retrieved for four plants, four fungi, eight invertebrates, four fishes, four non-mammalian tetrapods, two non-primate mammals, and four primates. The plants are *Zea mays* (zma), *Oryza sativa* (osa), *Vitis vinifera* (vvi), and *Arabidopsis thaliana* (ath). The four fungi are *Schizosaccharomyces pombe* (spo), *Saccharomyces cerevisiae* (sce), *Aspergillus nidulans* (ani), and *Ustilago maydis* (uma). The eight invertebrates are *Crassostrea gigas* (cgi), *Caenorhabditis elegans* (cel), *Daphnia pulex* (dpu), *Apis mellifera* (ame), *Atta cephalotes* (ace), *Aedes aegypti* (aae), *Anopheles gambiae* (aga), and *Drosophila melanogaster* (dme). The four fishes are *Danio rerio* (dre), *Salmo salar* (ssa), *Takifugu rubripes* (tru), and *Gadus morhua* (gmo). The four non-mammalian tetrapods are *Xenopus tropicalis* (xtr), *Anolis carolinensis* (aca), *Gallus gallus* (gga), and *Taeniopygia guttata* (tgu). The two non-primate mammals are *Bos taurus* (bta) and *Mus musculus* (mmu). The four primates are *Otolemur garnettii* (oga), *Pongo abelii* (pab), *Pan troglodytes* (ptr), and *Homo sapiens* (hsa). An image to illustrate each species was also retreived from the Ensembl resources. Taxonomic information for these species was downloaded from the NCBI Taxonomy resource Common Taxonomy Tree [24], and the phylogenetic tree was built with SeaView v5.0.5 [25].

### Data processing and representation

We looked for pure glutamine-coding stretches in the transcript datasets (CAA/CAG stretches), from length one codon, using an in-house Perl script. Per stretch, we annotated its length, sequence, number of CAA and CAG codons in it, codon usage, species, sequence ID, codons in positions −1 to −5 (counting from the start of the stretch), and codons in positions +1 to +5 (counting from the end of the stretch). Considering the complete set of glutamine-coding stretches per species, we used the number of CAA and CAG codons in them to calculate the species’ glutamine background codon usage. A length-specific codon usage was also calculated per stretch length per species. Using the length-specific codon usage, we generated one random stretch of identical length per found polyQ stretch, in order to have a random dataset of polyQ stretches with the same features as the one obtained from the proteomes.

We calculated the similarity between codons CAG and CAA and codons in positions −1 and +1 with respect to the polyQ stretch using the Hamming distance [26], counting the number of mismatched nucleotides at analogous positions of both codons.

Distributions were always pairwise compared using nonparametric Mann–Whitney *U* tests in R v4.4.2. The figures were produced with R v4.4.2 and packages ggplot2 v3.5.1, dplyr v1.1.4, patchwork v1.3.0, magick v2.8.5, ggpubr v0.6.0, scales v1.3.0, cowplot v1.1.3, rstatix v0.7.2, tidyr v1.3.1, and ggridges v0.5.6.

## Results and discussion

### Glutamine codon usage across eukaryotes

Amino acid codon usage is species-specific, and its variation depends mostly on taxonomy. Our aim in this work is to check whether polyglutamine codon usage follows common patterns across evolutionary diverse species. We downloaded the full set of transcripts for 30 eukaryotic models from a wide taxonomic range, encompassing four plants, four fungi, eight invertebrates, four fishes, four non-mammalian tetrapods, two non-primate mammals, and four primates.

We identified all glutamine-encoding stretches (stretches with CAA or CAG codons) from length one onward and annotated them with their length (Supplementary Table S1). All species show a comparable amount of glutamine stretches among other species from their same taxa, except *S. salar*. This fish has a disproportionate number of individual glutamines (length one), doubling the value for the human transcriptome, with a similar number of transcripts in the dataset. We have found in literature no explanation for this particular observation.

**Figure 1.**
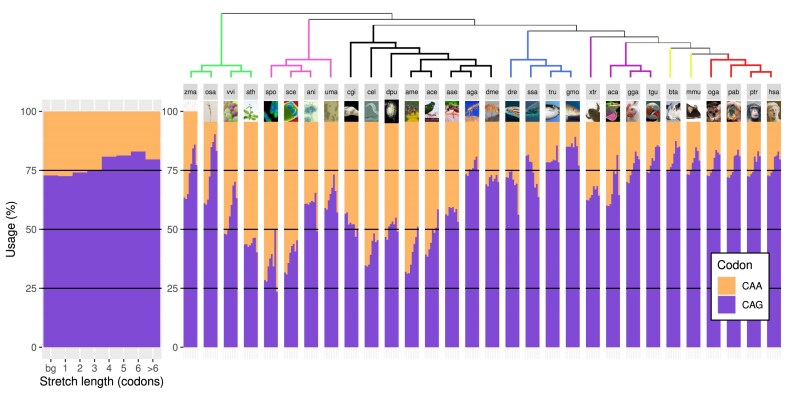
Glutamine codon usage in the complete transcriptome of 30 eukaryotic model species. The CAA (orange) and CAG (purple) codon usage are calculated independently per codon stretch length, plus an overall length-independent usage (bg). On the left, a zoomed representation of the plots in the right. On top, a phylogenetic tree relating the species, represented by a three-letter code (see the “Materials and methods” section). Colored branches represent plants (green), fungi (pink), invertebrates (black), fishes (blue), non-mammalian tetrapods (purple), non-primate mammals (lime), and primates (red).

Next, we studied the use of CAG and CAA codons in the glutamine stretches per specific stretch length, with the exception of stretches longer than 6 codons, grouped as length >6 codons. This was required because of the reduced number of stretches for longer lengths in some species. We also took all stretches together to calculate a background glutamine codon usage per species (annotated as “bg”) (Fig. 1).

Glutamine codon usage is taxonomically influenced but species-specific: while there is a trend for higher %CAG values in species taxonomically closer to humans, this is also valid for part of the most distant species, plants *Z. mays* and *O. sativa*. Similarly, even within fungi there is a high variability. A common trend between most of the species in the study is that longer stretches have higher %CAG values, independently of their background codon usage. This increase is higher when the background usage is lower; i.e. in *A. mellifera*, there is a 32% CAG usage in the background, and 53.2% CAG usage in stretches of six codons, while in *H. sapiens*, this increase is from 72.8% to 83%. The results obtained for human are in line with previously reported results [18].

An additional trend that can be seen in many species (23 out of 30) is that the longest stretches (>6 codons) have lower %CAG values than stretches of length 6 codons. This is also independent from background codon usage. Only one taxonomic group deviates from these trends, fishes, as they either decrease the %CAG with length, or maintain it stable. The number of long polyQ regions in fishes is comparable to their number in the human proteome (Supplementary Table S1).

### CAA codons are placed C-terminally to CAG codons in polyglutamine-coding regions

**Figure 2.**
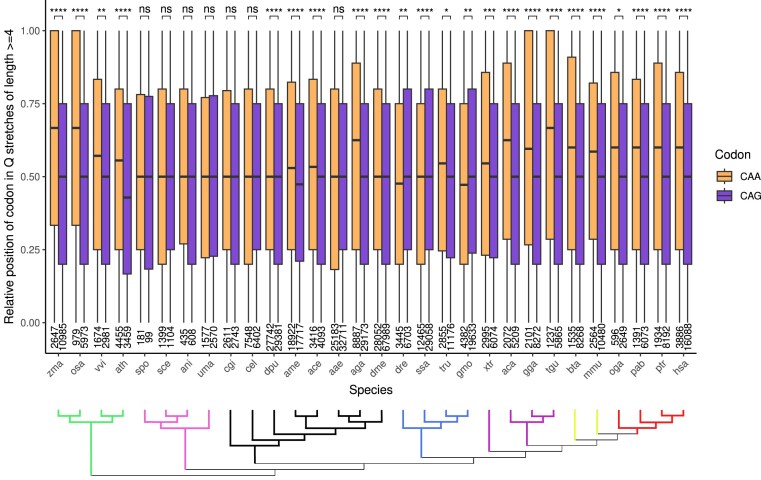
Mean relative position of codons in glutamine stretches of length ≥4 codons. The relative positions of the CAA (orange) and CAG (purple) codons are calculated with respect to each individual stretch length. The first codon maps to position = 0 (N-terminal part of the stretch), the last codon to position = 1 (the C-terminal part), and everything in between is evenly spaced. Nonparametric Mann–Whitney *U* statistical tests were performed to pairwise compare the distributions (***P-value <.001, **P-value <.01, ns = not significant). Below: a phylogenetic tree relating the species, represented by a three-letter code (see the “Materials and methods” section). Colored branches represent plants (green), fungi (pink), invertebrates (black), fishes (blue), non-mammalian tetrapods (purple), non-primate mammals (lime), and primates (red).

Since both CAA and CAG codons code for glutamine, theoretically there should not be any biases or differences in their positioning within a stretch translating to a polyQ region. In order to verify this, we calculated the distribution of the relative position of each codon in glutamine stretches of length ≥4, since shorter stretches would not be as informative (Fig. 2).

We described in the previous section how very long polyQ stretches (>6 codons) have in general a higher proportion of CAA codons than long stretches (6 codons). Here, we can see that CAA codons are positioned significantly more toward the C-terminal part of the stretches than CAG codons. This is true for plants, most invertebrates, and tetrapods, a total of 19 species out of the 30 studied. The reason for this behavior is proposed to be an internal mechanism within the polyQ stretch to stop the growth of the region via replication slippage (which more often affects CAG codons), through their mutation to CAA codons. However, different patterns are found in other taxonomies: in fungi, there are nonsignificant differences between the positions of the two codons, while in fishes CAA codons are placed more toward the N-terminal part of the stretch than CAG codons. This result could be an artifact related to the polyploidy in fishes. To assess whether this is affecting the results of our analysis, the four different salmon transcriptomes (*S. salar*) available in the Ensembl database release 113 were analyzed. While the number of cases varies between datasets, the overall results and trends remain similar (Supplementary Fig. S1).

### Codon mutation at polyglutamine flanking positions limit the growth of pure CAG stretches

Apart from the proposed internal mechanism in the polyglutamine region for mutating CAG codons into CAA codons, an external mechanism might also exist. Mutating a CAG codon to any other codon but CAA would shorten the polyQ region and prevent CAG stretches from being extended by a slippage event. To test this hypothesis, we analyzed the similarity between CAG or CAA codons to the codon in the first position after the polyQ region (position +1), in pure CAG stretches, pure CAA stretches, and mixed CAG/CAA stretches of length 2 codons or longer. To study this similarity, we used the Hamming distance between codon CA[GA] and codon +1, ranging from 1 to 3.

**Figure 3.**
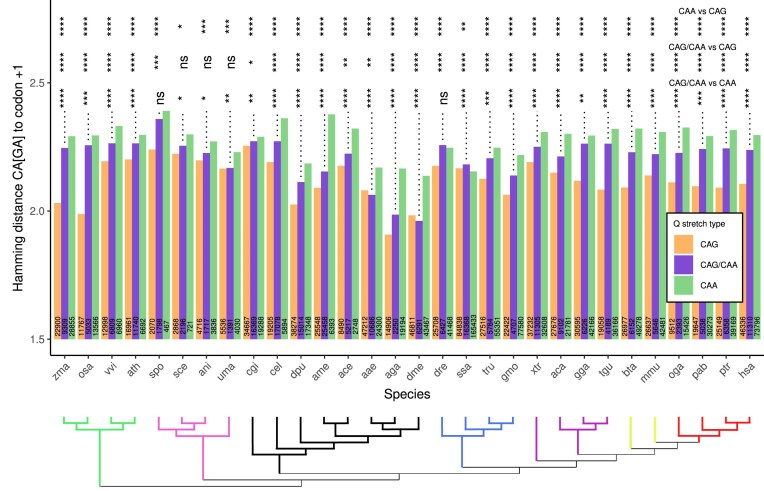
Hamming distance between CA[GA] codons to codon in position +1. The mean Hamming distance was calculated for pure CAG stretches (orange), mixed CAG/CAA stretches (purple), and pure CAA stretches (green). The number of cases considered per stretch type is shown at the bottom of the bars. Nonparametric Mann–Whitney *U* statistical tests were performed to pairwise compare the distributions from which the mean values were calculated. Comparisons are pure CAA versus pure CAG (top row), mixed CAG/CAA versus pure CAG (middle row), and mixed CAG/CAA versus pure CAA (bottom row) (***P-value <.001, **P-value <.01, *P-value <.05, ns = not significant). Colored branches represent plants (green), fungi (pink), invertebrates (black), fishes (blue), non-mammalian tetrapods (purple), non-primate mammals (lime), and primates (red).

Polyglutamine stretches encoded purely by CAG codons are significantly more similar to codon in position +1 than regions coding by CAA codons (Fig. 3). This is true for 29/30 species, all except the fish *S. salar*. Regions with mixed CAG/CAA codons have an intermediate Hamming distance. Results support the hypothesis that pure CAG stretches have more mutational events at their C-terminal region than pure CAA or mixed stretches, likely to shorten their length. It supports the hypothesis that CAA codons positioned C-terminally in the polyQ region are an efficient mechanism to stop the growth of polyQ stretches, hindering the CAG-promoted transcriptional slippage. The length of the region plays no role in their similarity to the codon in position +1; we repeated the analysis comparing different polyQ lengths and got similar results for all length ranges (data not shown).

**Figure 4.**
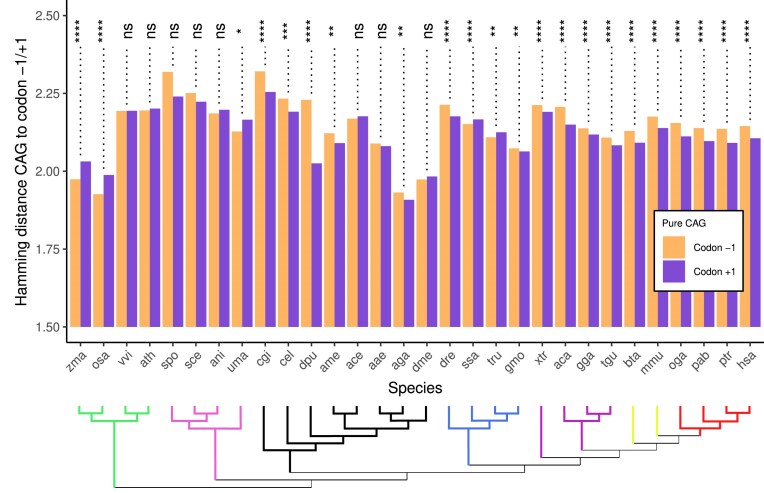
Hamming distance between CA[GA] codons to codons in positions +1 and −1, for pure CAG stretches. The mean Hamming distance was calculated in pure CAG stretches between CA[GA] and codons in position −1 (orange) and in position +1 (purple). Nonparametric Mann–Whitney *U* statistical tests were performed to pairwise compare the distributions from which the mean values were calculated (***P-value <.001, **P-value <.01, *P-value <.05, ns = not significant). Colored branches represent plants (green), fungi (pink), invertebrates (black), fishes (blue), non-mammalian tetrapods (purple), non-primate mammals (lime), and primates (red).

We focused on pure CAG regions and performed an analogous analysis but comparing the mean values of Hamming distance obtained when analyzing codons in positions −1 and +1 (the codons immediately preceding and following the polyQ stretches) (Fig. 4). All 10 species of Tetrapoda show lower values of Hamming distance in position +1 compared to position −1. In the rest of the species, this result is only significantly true for a few of them (7/20). These results show that the proposed mechanism of mutating CAG to nonsynonymous codons in order to control the length of the polyQ regions seems fixed in evolution from Tetrapoda.

### Amino acid context of polyglutamine stretches is taxa-dependent

The study of the codons close to the polyQ stretches has given us clues about mechanisms affecting the stretches themselves, even though we find them outside of the sequences. Here we extend this analysis to positions in the surrounding of the stretches, from position −5 to −1, and from position +1 to +5. We hypothesize that the amino acids in these positions do affect the polyQ stretches but at the structural/functional level, and not necessarily from the nucleotide level. In humans, amino acids enriched in the vicinity of homorepeats are also enriched compared to the amino acid usage of the complete proteome [27].

**Figure 5.**
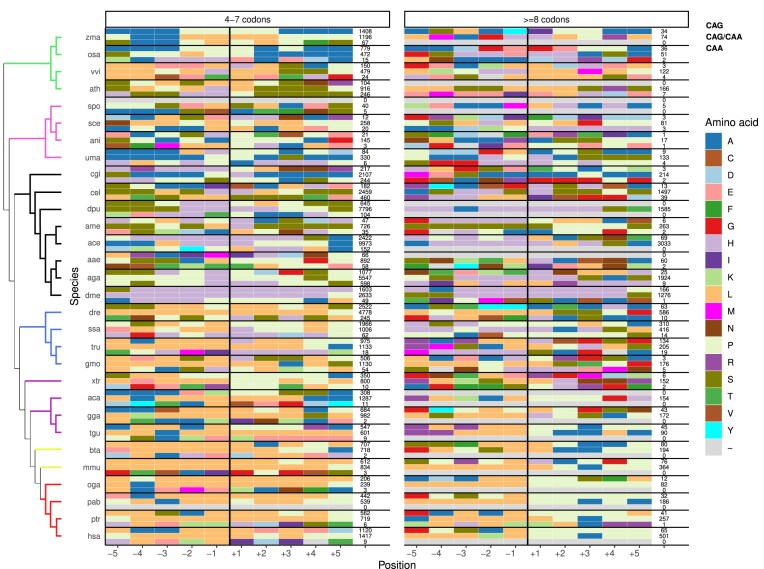
Top enriched amino acid in the surrounding polyglutamine stretches. Top enriched amino acid, not taking into account glutamine, in polyglutamine stretches of length 4–7 codons and ≥8 codons. Per species, stretches are distributed depending on whether they are coded by pure CAG (top row), mixed CAG/CAA codons (middle row), or pure CAA (bottom row). The number of stretches considered per condition is shown to the right of the row. The color of the cell represents the top enriched amino acid per position. Missing values are annotated as “–”. For the value of the enrichment, see Supplementary Fig. S2. Colored branches represent plants (green), fungi (pink), invertebrates (black), fishes (blue), non-mammalian tetrapods (purple), non-primate mammals (lime), and primates (red).

We measured the amino acid usage at each position surrounding polyQ stretches of 4–7 codons and ≥8 codons in all species, to analyze whether there are shared patterns and whether they change with stretch length. To do so, we sum the most popular amino acid per position. Then, we grouped the sequences by codon composition, in pure CAG, mixed CAG/CAA, and pure CAA, to check whether these patterns depend on the composition of the stretch (Fig. 5 and Supplementary Fig. S2).

Most positions around the polyglutamine stretches are glutamine residues, indicating that they tend to be either in glutamine-rich regions or in longer impure glutamine stretches (not depicted in Fig. 5; Supplementary Fig. S2). In Fig. 5, we leave out glutamines so, we can see which other amino acids appear in or near polyQ stretches and whether any pattern influences their structure and/or function. The amount of pure CAA stretches in tetrapods is very low; thus, no significant conclusions can be drawn for these regions. Tetrapods do also share a common leucine enrichment in most positions, probably given by chance (leucine is the most enriched amino acid in their proteomes). However, long polyQ stretches in tetrapods are enriched in proline residues in C-terminal positions. This pattern has been previously described to reduce the polyQ propensity to aggregate [14], by forming PPII helices and therefore decreasing the probability of the polyQ region to form a β-rich state [15].

Proline residues are coded by codons CC[ACGT]. One may argue that the presence of proline residues in positions following long polyQ stretches influences the previous result discussed in the “Codon mutation at polyglutamine flanking positions limit the growth of pure CAG stretches” section as for the similarity of codon +1 to CAG in pure CAG regions. However, as stated previously, that result is not influenced by the length of the region, whereas the proline enrichment following the polyQ is observed in long pure CAG or mixed regions.

Tetrapods do also have a shared alanine residue in position +3 of mixed CAG/CAA polyQ regions. Polyproline regions, the context in which this alanine residue is found, form PPII helices. It has been described that in PPII, a small residue (glycine) may appear in every third position to ease steric clash and preserve the helix shape, letting the chain fold in a stable, proline-rich structure [28]. Our hypothesis is that the alanine residue may be performing the same function here, since it is also a small amino acid.

Positions surrounding polyQ stretches in invertebrates are enriched in histidine residues. The reason behind this trend is unknown, but it is species-, composition-, and length-independent. We believe the presence of histidine residues is explained by its coding, as it is coded by CAC and CAT, similar codons to the ones coding for glutamine. Fishes show an intermediate behavior between invertebrates and tetrapods, with leucine enriched N-terminally to the polyglutamine stretches, and proline and histidines C-terminally.

### The maximum number of consecutive identical codons is driven by chance

For a polyglutamine region of length five amino acids in a proteome with a codon usage of 75% CAG/25% CAA (i.e. mammals; see Fig. 1), the random probability that the longest stretch of consecutive CAG codons in this region is of length three is 23.73%, of length four is 15.82%, and not having any CAA is 23.73%. For a region of six amino acids, these probabilities are 26.37% (length three), 17.80% (length four), 11.86% (length five), and 17.80% (length six). If the codon usage is 50% CAG/50% CAA, as in *D. pulex*, the random probabilities are 15.63%, 6.25%, and 3.125% for a region of length five, and 18.75%, 7.81%, 3.12%, and 1.56% for a region of length six. We wanted to test whether there is any type of pattern in which CAA codons (usually the less common codons in polyQ stretches) are placed at fixed positions with respect to CAG codons, impeding CAG to reach a maximum number of identical consecutive codons comparable to the one expected by its usage.

**Figure 6.**
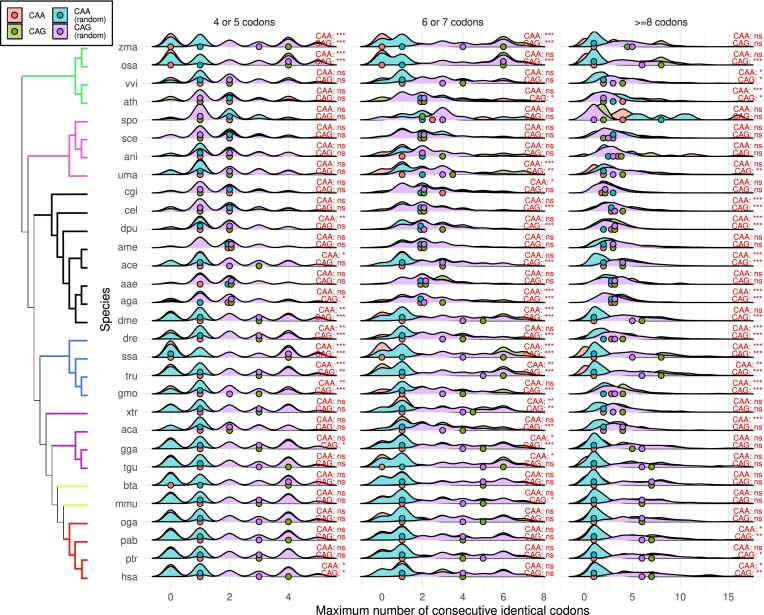
Distribution of the maximum number of consecutive identical codons in polyglutamine stretches. Per species (left), represented by a three-letter code (see the “Materials and methods” section), distribution of the maximum number of consecutive CAA (red) and CAG (green) codons in stretches of length 4 or 5, 6 or 7, and ≥8 codons. Sets of polyglutamine stretches with identical length distributions were generated randomly, and the distributions were also calculated for them (CAA random, in blue; CAG random, in purple). Nonparametric Mann–Whitney *U* statistical tests were performed to pairwise compare the distributions CAA versus CAA_random and CAG versus CAG_random (***P-value <.001, **P-value <.01, *P-value <.05, ns = not significant). The median value per distribution is depicted with a filled circle. Colored branches represent plants (green), fungi (pink), invertebrates (black), fishes (blue), non-mammalian tetrapods (purple), non-primate mammals (lime), and primates (red).

We calculated the maximum number of consecutive identical codons, for both CAA and CAG codons, in regions of 4 or 5 codons, 6 or 7 codons, and ≥8 codons. This was done independently per species. We also produced sets of randomly generated sequences, with the same length distributions, by using the previously calculated length-specific codon usage (see the “Materials and methods” section). The difference between the observed maximum number of consecutive identical codons and the expected when compared to random regions is not significant in the majority of the conditions (length, codon, and species) (Fig. 6). Teleostei (fishes: ssa, gmo, tru, dre) is the only taxa in which most of the comparisons with the random condition are significant: higher number of consecutive CAG codons and fewer number of CAA codons than expected by chance (only 2/24 conditions are not significant).

There seems to be no common mechanism for which the inclusion of CAA codons in polyQ stretches avoids the growth of pure CAG stretches to their expected length by chance, which is directly related to the length-specific codon usage per species.

### Codon patterns in polyglutamine stretches

We have previously described a trend for CAA codons to be positioned C-terminally in polyQ stretches. Not accounting for this trend, it is expected for codons to be distributed randomly within these regions. To check whether there are differences in the distribution of codon patterns within mixed CAG/CAA polyQ regions, we have calculated the relative amount per codon pattern composed of four codons. To simplify the interpretation of the patterns, CAG codons are replaced by “G” and CAA codons by “A.” For regions longer than four codons, all possible four-codon stretches were accounted for; e.g. region G·G·G·G·G·A·G is composed of patterns G·G·G·G, G·G·G·G, G·G·G·A, and G·G·A·G. While the codon usage of CAG versus CAA plays a role in the relative proportion among the different patterns, those with the same amount of CAG and CAA codons are free from this bias; i.e. G·G·A·A can be compared with G·A·G·A, A·A·G·G, and A·G·A·G.

**Figure 7.**
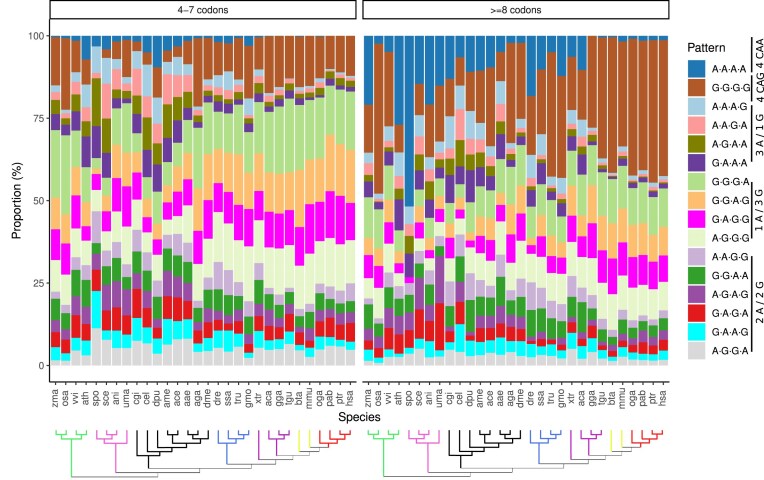
Pattern distribution of quadruplets in mixed CAG/CAA polyglutamine stretches. Proportion of all possible four-codon stretches within polyglutamine stretches of 4–7 codons and ≥8 codons. For clarity in the representation of the patterns, codon CAG is replaced by “G” and codon CAA by “A”; i.e. pattern CAACAACAACAA is replaced by A·A·A·A. Colored branches represent plants (green), fungi (pink), invertebrates (black), fishes (blue), non-mammalian tetrapods (purple), non-primate mammals (lime), and primates (red).

The length of the polyQ region from which the four-codon stretch is obtained determines the distribution of the CAG/CAA patterns (Fig. 7). In species without a deep length-dependent codon usage, such as *T. rubripes*, this should not be the case. However, for this species, pattern G·G·G·G is three times more prevalent in longer stretches (37.8% versus 12.6%). It is intriguing that, also in *T. rubripes*, with a constant CAG usage of 78% (but in regions of length 6 codons, with 85%), there is a 7.4-fold increase in the proportion of pattern A·A·A·A in longer stretches (4.97% versus 0.67%). This increase is shared by the rest of the fishes in our study, as well as by plants, fungi, and even some invertebrates considered. Pattern A·A·A·A is, on the other hand, depleted in tetrapods. This behavior is the opposite to what we previously reported about prolines appearing C-terminally to the polyQ regions in Tetrapoda (see the “Amino acid context of polyglutamine stretches is taxa-dependent” section), indicating that there may be two analogous mechanisms for limiting the aggregation propensity of the CAG codons, both dependent on the stretch length.

Mixed CAG/CAA patterns with codon proportion 3-to-1 share in general the same prevalence among those with identical codon composition. Exceptions are A·A·A·G and G·A·A·A, generally more prevalent than A·A·G·A and A·G·A·A, but depending on the species. Patterns with three CAG codons and one CAA are four to five times more prevalent than pure CAG stretches in shorter regions, but are similarly enriched in longer regions. This is in line with what was described before about CAG codons being responsible for the longest polyglutamine regions, even in species with lower CAG usage. The six patterns with two CAG and two CAA codons do not show any remarkable difference in proportion.

## Conclusions

Our findings reveal that taxonomically diverse species share general patterns of glutamine codon usage. We detected a clear tendency in most species to increase their proportion of CAG codons in longer polyQ stretches. This pattern, however, is not found in Teleostei (fishes), which either reduce or maintain stable CAG usage as stretch length grows. Our data suggest that multiple factors influence the codon choice in polyQ regions, including background codon frequencies, replication slippage, and structural constraints. An additional factor to take into account that may influence the codon choice in polyQ regions is transfer RNA (tRNA) availability. The human genome has roughly 30 copies of the tRNAGlnCUG gene that matches CAG but only half as many of the tRNAGlnUUG gene that matches CAA [29]. Because silent changes have a small evolutionary cost and mammals have low effective population sizes, even mild gains from translation speed can be quickly fixed in evolution.

We found that CAA codons often cluster closer to the C-terminal side of polyQ stretches in plants, invertebrates, and tetrapods. We propose this shift in codon position may act as an internal mechanism that interrupts further expansion of pure CAG repeats, thus slowing potential slippage-driven growth. Fungi do not show significant differences in codon position, and fishes show an opposite pattern. These differences highlight the strong taxonomic influence on codon choice and position.

Flanking codons may also restrain CAG stretches. In many tetrapods, the codon immediately following a pure CAG stretch tends to be more similar in sequence, suggesting that these regions have experienced mutational events that switch CAG to similar codons encoding other amino acids. This might act as an external mechanism to limit CAG expansions. Flanking amino acids reveal another layer of complexity: tetrapods often place proline and alanine residues, known to reduce the β-rich aggregation of long polyQ repeats, on the C-terminal side. Invertebrates prefer histidine residues near these stretches, perhaps influenced by the codon similarity between histidine and glutamine rather than structural needs.

Regardless of these patterns, the maximum run of consecutive identical codons often fits random expectations. Thus, even though internal and external mechanisms exist to influence codon use and position, chance remains a strong driver of how polyQ regions evolve. Only Teleostei deviate from random, showing more consecutive CAG stretches than expected, along with fewer consecutive CAA stretches.

Taken together, our results highlight that both local (codon usage, structural concerns, and mutational events) and global (taxonomic constraints and random processes) factors guide the formation and placement of CAA and CAG codons in polyQ stretches. These findings expand our understanding of how repetitive stretches behave across diverse eukaryotes, suggesting the convergence of distinct evolutionary paths and cellular processes to produce similar outcomes in different groups.

## Supplementary Material

lqaf075_Supplemental_File

## Data Availability

The data underlying this article are available in the article and in its online supplementary material and uploaded in the Zenodo repository (doi:10.5281/zenodo.15310501).
