# Supplementary material for: Evolutionary perspective of the CAG/CAA interplay coding for pure polyglutamine stretches in proteins
Source: NAR Genom Bioinform. 2025 Jun 9;7(2):lqaf075. doi: 10.1093/nargab/lqaf075 (PMC12147016; doi:10.1093/nargab/lqaf075)
Supplement: lqaf075_Supplemental_File [file lqaf075_supplemental_file.docx]

| **Species \ Length** | **1** | **2** | **3** | **4-5** | **6-7** | **>=8** |
| --- | --- | --- | --- | --- | --- | --- |
| **zma** | 934006 | 52461 | 5779 | 2249 | 467 | 108 |
| **osa** | 522785 | 26344 | 2647 | 1021 | 263 | 91 |
| **vvi** | 511138 | 24092 | 1878 | 541 | 127 | 129 |
| **ath** | 668836 | 31139 | 2798 | 1004 | 279 | 173 |
| **spo** | 82145 | 4084 | 200 | 38 | 8 | 5 |
| **sce** | 99305 | 4829 | 576 | 222 | 68 | 90 |
| **ani** | 182369 | 9303 | 773 | 150 | 23 | 20 |
| **uma** | 151799 | 9377 | 1007 | 340 | 86 | 147 |
| **cgi** | 1238347 | 64291 | 4941 | 894 | 136 | 62 |
| **cel** | 553455 | 34920 | 4466 | 2113 | 457 | 221 |
| **dpu** | 741098 | 52819 | 8618 | 5006 | 2233 | 1960 |
| **ame** | 714142 | 46419 | 6315 | 2156 | 952 | 1557 |
| **ace** | 225193 | 13829 | 1543 | 584 | 226 | 273 |
| **aae** | 773520 | 60174 | 11872 | 6348 | 2217 | 1587 |
| **aga** | 330357 | 25931 | 4667 | 2913 | 1391 | 1448 |
| **dme** | 836170 | 67604 | 13257 | 9147 | 3453 | 3107 |
| **dre** | 1116157 | 65918 | 5805 | 1369 | 327 | 184 |
| **ssa** | 4062119 | 234434 | 23982 | 6675 | 888 | 659 |
| **tru** | 1340677 | 78940 | 7224 | 1793 | 336 | 358 |
| **gmo** | 1688052 | 92874 | 8042 | 2352 | 700 | 741 |
| **xtr** | 1302188 | 73656 | 5726 | 1397 | 212 | 154 |
| **aca** | 919062 | 53194 | 4037 | 956 | 212 | 160 |
| **gga** | 1183386 | 70733 | 6363 | 1417 | 259 | 215 |
| **tgu** | 896430 | 53377 | 4732 | 950 | 219 | 135 |
| **bta** | 1361089 | 74643 | 6057 | 1173 | 260 | 274 |
| **mmu** | 1185500 | 67185 | 5687 | 1149 | 303 | 440 |
| **oga** | 451298 | 24699 | 2083 | 378 | 76 | 95 |
| **pab** | 875940 | 49520 | 4232 | 801 | 187 | 218 |
| **ptr** | 1121569 | 63606 | 5473 | 1031 | 267 | 299 |
| **hsa** | 2079812 | 118170 | 10133 | 2108 | 465 | 566 |

**Supplementary Table 1.** Number of glutamine stretches per length and species.


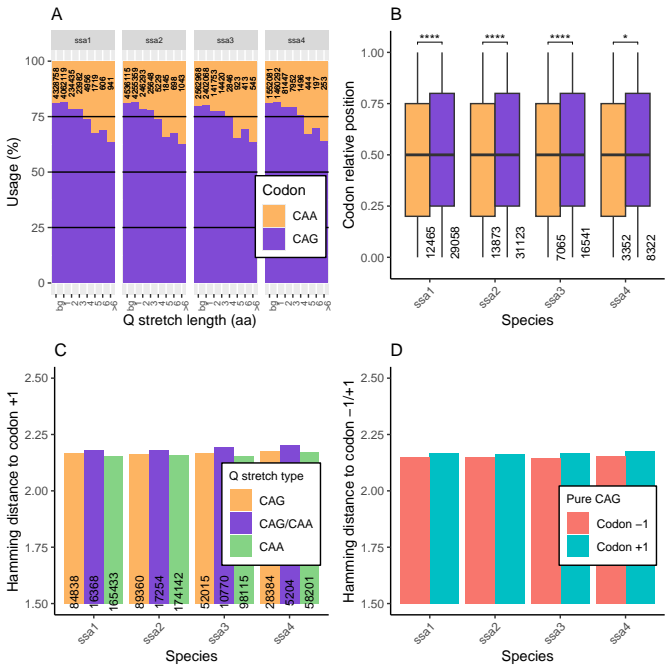
**Supplementary Figure 1.** Analysis of the glutamine codon usage (A), codon relative position within the polyglutamine regions (B), Hamming distance to codon +1 in pure CAG, pure CAA and mixed CAG/CAA stretches (C), and Hamming distance to codons +1 and -1 in pure CAG stretches (D), in transcriptomes of four *Salmo salar* assemblies (ssa1 = Ssal_v3.1, ssa2 = Ssal_ALTA, ssa3 = USDA_NASsal_1.1, ssa4 = Ssal_Brian_v1.0).


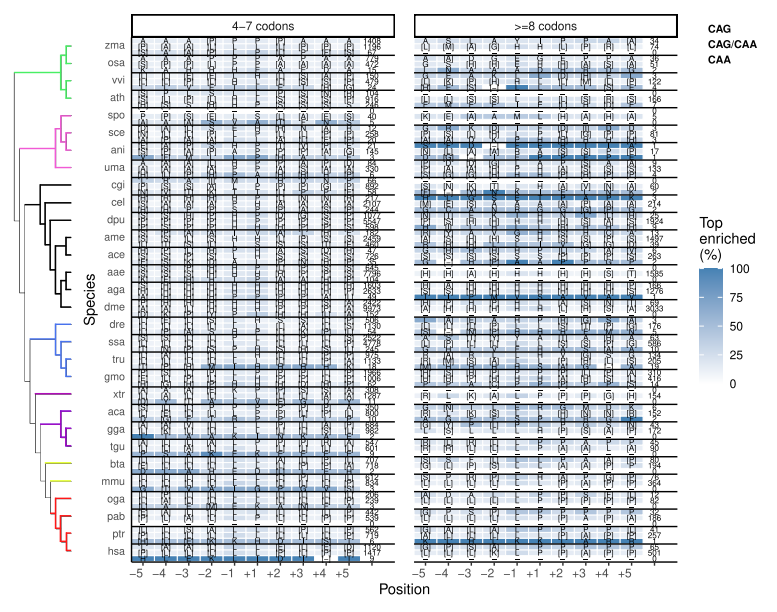
**Supplementary Figure 2.** Top enriched amino acids in the vicinity of pure CAG, mixed CAG/CAA and pure CAA regions; amino acids are shown in brackets if glutamine is the top most enriched amino acid and they are the second most enriched.
